# Our Faces in the Dog's Brain: Functional Imaging Reveals Temporal Cortex Activation during Perception of Human Faces

**DOI:** 10.1371/journal.pone.0149431

**Published:** 2016-03-02

**Authors:** Laura V. Cuaya, Raúl Hernández-Pérez, Luis Concha

**Affiliations:** Instituto de Neurobiología, Universidad Nacional Autónoma de México, Querétaro, México; University Of Cambridge, UNITED KINGDOM

## Abstract

Dogs have a rich social relationship with humans. One fundamental aspect of it is how dogs pay close attention to human faces in order to guide their behavior, for example, by recognizing their owner and his/her emotional state using visual cues. It is well known that humans have specific brain regions for the processing of other human faces, yet it is unclear how dogs’ brains process human faces. For this reason, our study focuses on describing the brain correlates of perception of human faces in dogs using functional magnetic resonance imaging (fMRI). We trained seven domestic dogs to remain awake, still and unrestrained inside an MRI scanner. We used a visual stimulation paradigm with block design to compare activity elicited by human faces against everyday objects. Brain activity related to the perception of faces changed significantly in several brain regions, but mainly in the bilateral temporal cortex. The opposite contrast (i.e., everyday objects against human faces) showed no significant brain activity change. The temporal cortex is part of the ventral visual pathway, and our results are consistent with reports in other species like primates and sheep, that suggest a high degree of evolutionary conservation of this pathway for face processing. This study introduces the temporal cortex as candidate to process human faces, a pillar of social cognition in dogs.

## Introduction

Faces provide us with valuable information about others, e.g., species, age, gender, motivational and attentional state. There is evidence from behavioral, neuropsychological and neurophysiological studies to suggest the existence of a specialized mechanism to process the perception of faces [1]. In humans, this mechanism is located in the temporal cortex, specifically in the fusiform face area [2], and fMRI studies have shown that this cerebral region responds at least twice as strongly when the participant observes faces in comparison to other classes of visual stimuli [3]. Electrophysiological studies have found that non-human primates have an anatomically and functionally equivalent region required for face perception called the inferotemporal region [4]. However, the processes involved in face perception and discrimination are not the same in all species. For example, Japanese monkeys (*Macaca fuscata*) are incapable of naturally discriminating between two human faces [5], even though they are able to discriminate between two Japanese monkey faces. Dogs (*Canis familiaris*), on the other hand, are perfectly adept at an equivalent task.

Unlike non-human primates, dogs are an excellent model to study the social cognition in a comparative approach, as they possess unique cognitive skills that make them more similar to a human infant than other species [6–10]. Although the ability to discriminate between two human faces is not exclusive to dogs (it has been observed in other species that are in close contact with humans, such as sheep), the detail of the information that a dog can acquire from a mere glimpse towards a human face, even without training [11], is extraordinary. Dogs are especially good at discriminating between two humans, even if they are both familiar to them [12], but also, they have a remarkable ability to pick up small but important signals in a human face, like the attentional state [13] (e.g., they prefer to ask for food from a human with whom they can establish eye contact), and the emotional state [14] (they can discriminate between smiling and neutral faces). Similarly, dogs spend more time looking at a new human face in an image than at a familiar one, which suggests that they can discriminate between individuals using only visual cues [15]. Also, dogs pay significantly less attention to their owner, if the owner has his or her head covered [16]. This tendency to look at a human face during interaction has not been found in other canids, not even in extremely socialized wolves [11]. Altogether, these findings show that dogs are capable of perceiving subtle traits in human faces and that they use this information to modulate their behavior.

Nevertheless, the canine cerebral correlates of face perception have not been addressed. Functional magnetic resonance imaging (fMRI) is a valuable tool to assess regional cortical activity, and has recently been successfully applied in dogs to study auditory [17], visual [18,19] and olfactory [20] processes. In this work we use fMRI to describe the cerebral correlates of human face perception in dogs. This will lead us closer to understanding the underlying cerebral activity of one of the foundations of the dog's social cognition. Our results show several brain regions involved in the processing of faces by dogs, including the bilateral temporal cortex, right frontal cortex, medial frontal cortex, thalamus and caudate nucleus.

## Methods

### Participants

Seven healthy dogs participated in the study (four neutered males and three females; ages 15 to 50 months). The sample included five Border Collies, one Labrador Retriever and one Golden Retriever, all recruited through local families. The main caretaker of each dog gave informed consent and the dogs lived with their human families throughout the study. All procedures were performed in compliance with international ethics and animal care guidelines, and the study was approved by the Bioethics Committee of the Institute of Neurobiology, Universidad Nacional Autónoma de México (File 26RM).

### Training

**Fig 1:**
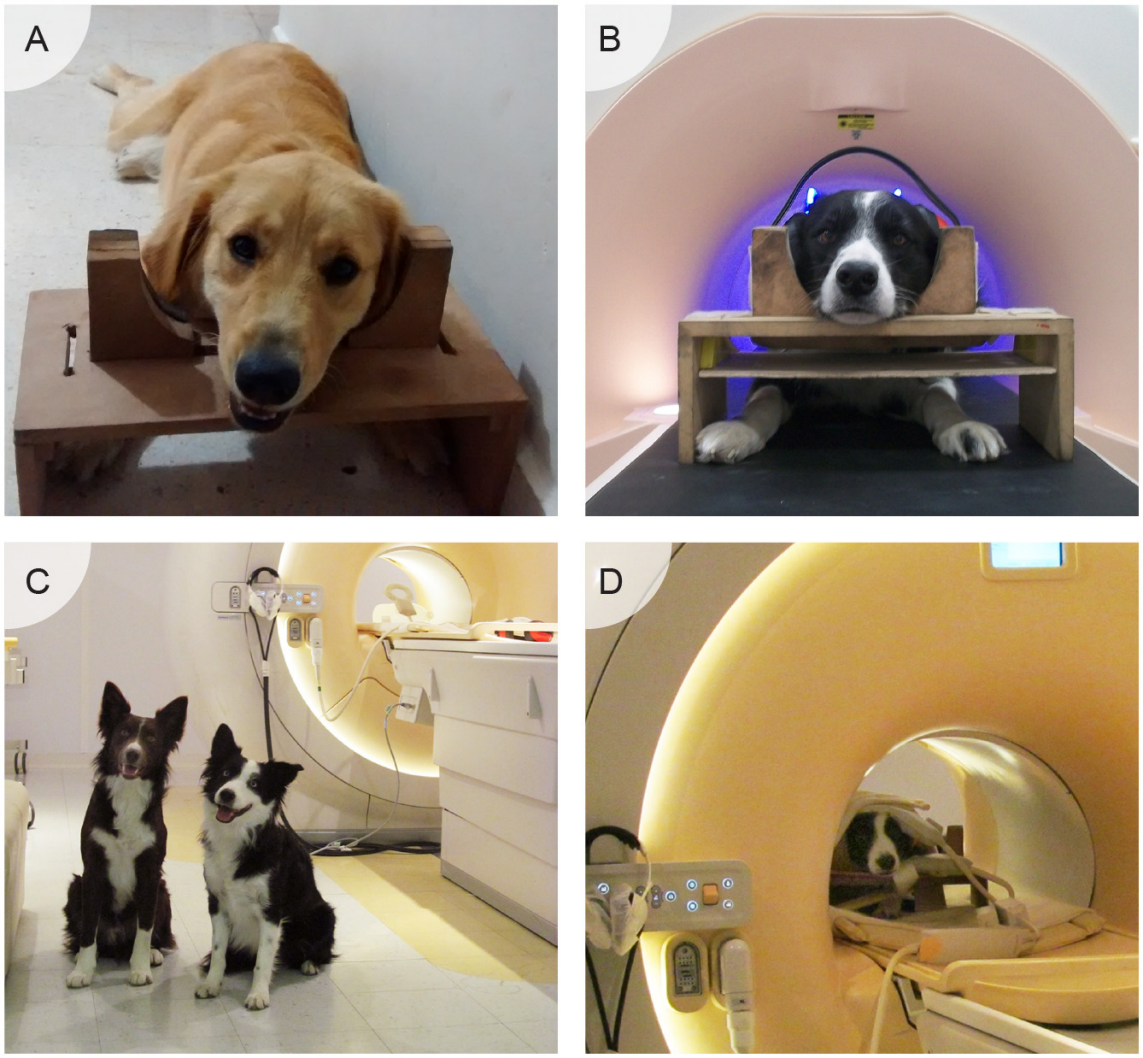
Training procedure for awake dog fMRI. During a period of approximately four months, dogs learned to stay still and attend to a visual stimulation paradigm within the MRI scanner. First, dogs learned to rest their chin and avoid movement (**A**), followed by habituation to the scanning environment, including scanner sounds and the use of protective headphones, in a mock MRI scanner (**B**). Next, dogs were further trained within the real MRI scanner (**C**) and habituated to the imaging coils (**D**). Upon completion of training, all dogs were able to lie still and awake for periods of up to 15 minutes.

All the dogs completed training prior to the imaging sessions, with the goal being that the dogs remain still inside the scanner while watching images projected during the acquisition of fMRI (Fig 1). To accomplish this, we first trained the dogs to remain still in sphinx position, with their head supported in a chin rest similar to the one reported in other studies [18]. The chin rest used is adjustable to different dog head sizes, and it helped the dogs maintain the sphinx position. The first part of the training was performed in the dog owner’s house until the dog was able to stay still for five minutes. The next step consisted of training the same behavior in a mock scanner. This part of the training included sounds similar to those produced by the real scanner, the use of noise protectors for dogs and staying still without seeing humans (to prepare for the projection of images). Finally, the last training step was inside the real scanner, adding the positioning of the MRI coil and projection of visual stimuli.

The three training stages were conducted under the same basic principles: at the beginning the dogs habituated to the situation and its new elements; all training sessions lasted a maximum of 15 minutes—if there were multiple sessions in a day, they were interspersed with periods of rest and play. The frequency of training sessions was, on average, once per week. We used shaping, chaining and positive reinforcement to train the behavior, and the dogs could leave the session at any time.

### Stimuli

As stimuli, we used photographs of 50 human faces of both genders with a neutral expression extracted from the AR Face Database [21] (with the author's permission), and 50 everyday objects. All face images were presented in a frontal view. In order to have more natural stimuli, all photographs were in color. The images of everyday objects were original photographs. All photographs were taken under the same lighting conditions, were edited to maintain a similar size, and were adjusted to the same brightness and contrast conditions as the AR Face Database photographs. Images were back-projected to a screen in front of the dogs, at a distance of 1.5 m (the maximum distance for a dog to be able to discriminate details on a face is close to six meters [12]). In order to preserve a natural appearance for the stimulus, the size of the projected images was similar to that of a real face (15 cm × 20 cm), as this size has been found to be adequate in other studies [12,14]. Each stimulus was presented only once in each run to avoid habituation to the images.

**Fig 2:**
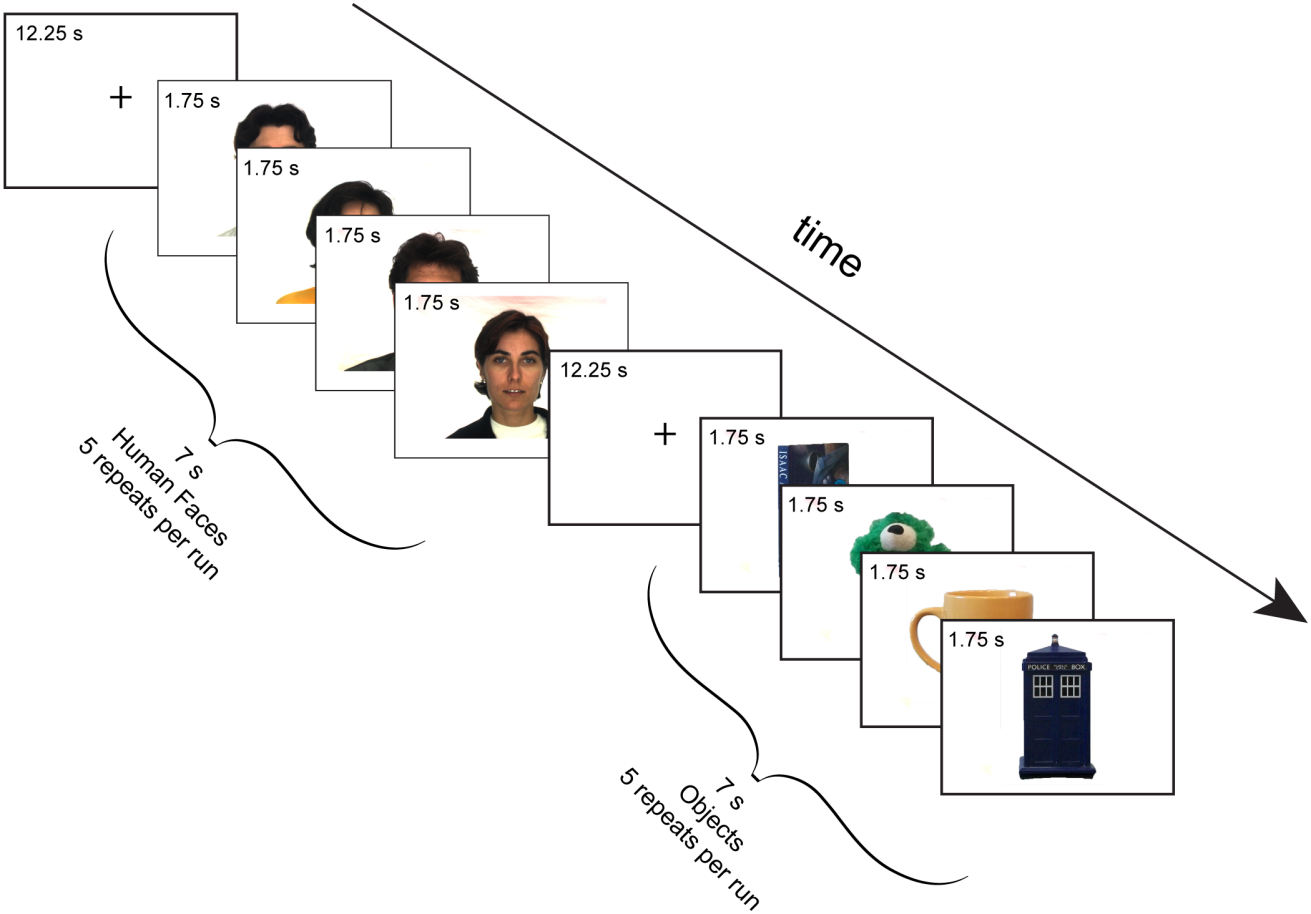
Visual stimulation paradigm. We used a block paradigm constituted by two types of blocks: Human faces and Objects. Each block was presented for 7 s, and it was composed of 4 different images (i.e., each image was visible for 1.75 s). At the beginning of each run and after each block, a fixation screen was presented for 12.25 s. The total duration of each run was 190 s, and each dog experienced 5 runs in total. Photographs of faces are reprinted from the AR face database [21] under a CC BY license, with permission from the author.

The visual stimulation paradigm had a block design, with each block lasting 7 s and including 4 images (Fig 2). There were two types of blocks: unfamiliar *faces* and everyday *objects*; blocks were separated by 12.25 s. The order of the blocks in each run was pseudo randomized. Each fMRI run had duration of 190 s and included 10 blocks (5 of human faces and 5 of everyday objects). Each participant observed 5 runs; in each run the types of block were presented in a different order. Visual stimulus presentation was controlled by PsychoPy2 [22]. The lights in the MRI suite were turned off during paradigm presentation. An experimenter was present in the MRI suite (out of the dog’s sight) during image acquisition to ensure the dog’s compliance with the task. To maximize compliance, only 1–3 runs were acquired per imaging session, with the dogs returning on subsequent days, until 5 runs were acquired successfully.

### Data acquisition

All images were acquired at the Institute of Neurobiology at the Universidad Nacional Autónoma de México. We used a 3 T Philips Achieva TX scanner equipped with a SENSE Flex Small coil which consists of two circular elements of 11 cm in diameter, one placed above the cranium and the second underneath the muzzle, both secured with velcro. This kind of coil was successfully used in a previous study, and it has the advantage of being able to fit any dog’s head size [17]. The blood oxygen level dependent (BOLD) images covered the whole brain with 28 contiguous coronal slices acquired with a gradient-echo echo-planar imaging (EPI) sequence (TR/TE = 1750/30 ms; flip angle = 90°; FOV = 224×240 mm^2^, acquisition matrix 112×120, resulting in 2×2×3 mm^3^ spatial resolution). Each run included 110 volumes and five dummy scans. Also, a standard T1-weighted structural image with a turbo spin echo sequence with 75 slices covering the whole brain (with 1×1×1 mm^3^ spatial resolution) was acquired for anatomical reference and registration.

### Image Analysis

All functional images were preprocessed for temporal and motion correction using FSL [23] version 4.19. The brain was extracted using manual segmentation. Spatial smoothing was performed using a Gaussian kernel with FWHM = 7 mm. Motion correction was performed separately on each run. We discarded runs that showed motion throughout the scan, as defined by head rotation greater than 1° or translation greater than 3 mm [17]. Considering all sessions, less than 20% of functional images were discarded. Functional images were spatially normalized to a digital atlas of the dog's brain [24].

Statistical analyses of fMRI data were performed using the General Linear Model as implemented in FSL. The regressors in this model included stimuli vectors of human faces and everyday objects. Regressors were convolved with the canonical hemodynamic response function modeled as a Gamma function. First, each run was analyzed individually in a first level. Next, for each participant, we made a single subject fixed effect analysis with five runs. At the group level we made a random effects analysis with the seven participants. In order to describe the cerebral regions involved in the perception of human faces, we analyzed the contrasts faces vs. objects in a whole-brain, voxel-wise analysis. The resulting statistical parametric maps were threshold using random field theory [25] (thresholded at z > 2.2 and p_cluster_ < 0.05). To localize and label the cerebral structures, we used two atlases: the digital atlas of Datta et al. [24] that is derived from fifteen mesocephalic dogs (this category includes breeds similar to the ones used herein) and the Beagle Brain in Stereotaxic Coordinates of Palazzi [26]. Coordinates reported refer to the Datta atlas.

We created a sphere of 5 mm radius around the voxel with the maximum *z* value to extract the BOLD signal change. The BOLD responses for faces and objects derived from this spherical region of interest were compared using a two tailed paired t-test and differences were deemed significant if p < 0.05.

## Results

The time to complete the training varied among participants because the frequency of the sessions differed and ranged from 1 to 4 months. The experimenter inside the scanner room visually monitored the dogs to make sure they were awake and attentive. As maintenance of the sphinx position requires muscle tone, loss of which would result in major head movement, the criteria used for motion correction of imaging data further guarantee that the dogs remained awake.

**Fig 3:**
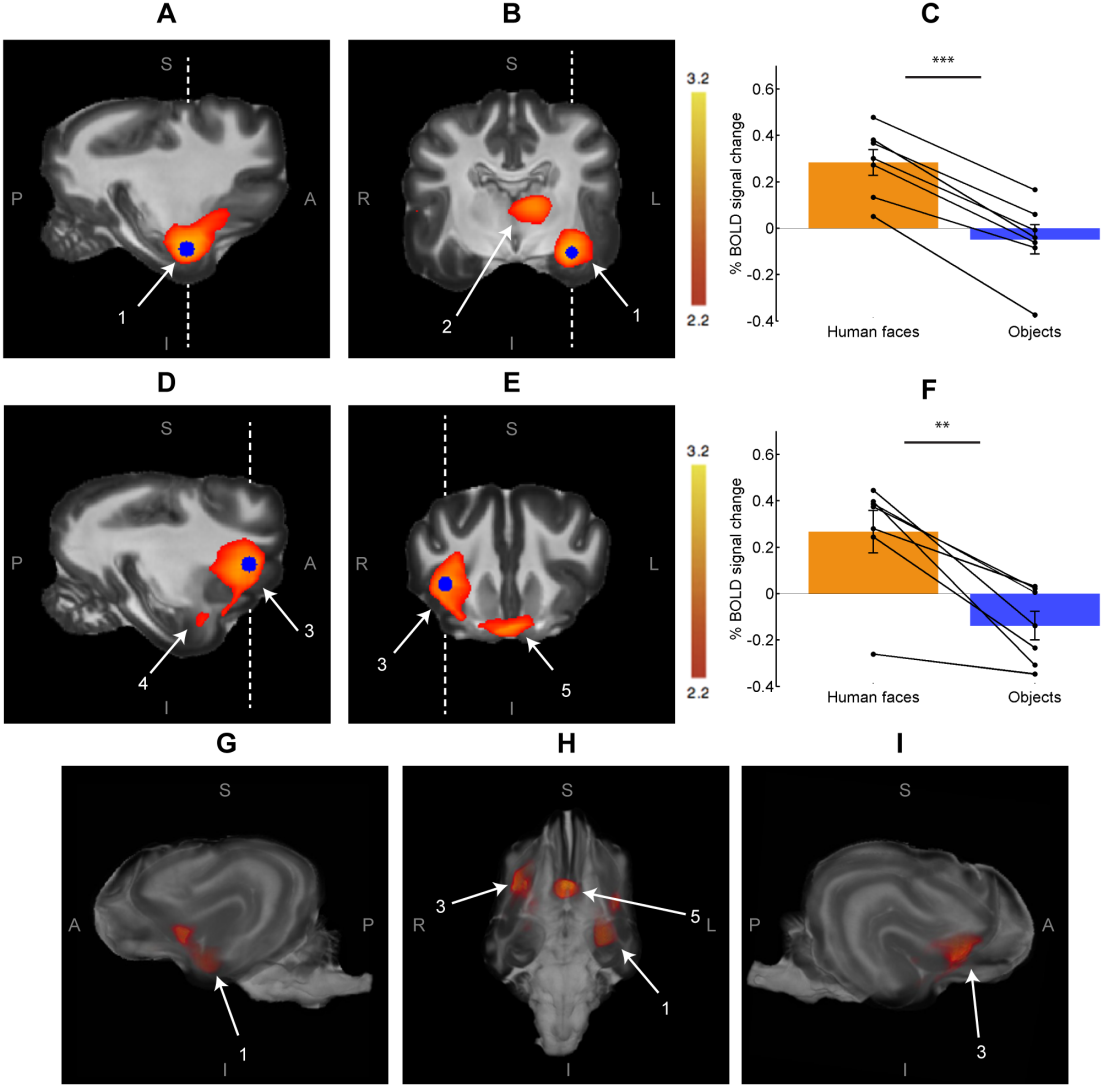
Human faces > Objects (n = 7). The two largest resulting clusters are shown overlaid on the Datta atlas [24]. **A.** Sagittal slice of the temporal cluster, which shows activity within the left temporal and frontal cortices. **B.** Coronal slice shows the left temporal cluster, which extends to the thalamus. **C.** BOLD signal change in a 5 mm of radius sphere around the voxel with maximum *z* value for the left temporal cluster (blue sphere in A and B). **D.** Sagittal slice of the frontal cluster, which is located in the right frontal temporal and cortices. **E.** Coronal slice of the frontal cluster. **F.** BOLD signal change in a 5 mm of radius sphere around the voxel with maximum *z* value for the right frontal cluster (blue sphere in D and E). **G-I.** Volume renderings of the same results, seen in left lateral, basal and right lateral views, respectively. S = Superior, I = Inferior, L = Left, R = Right, P = Posterior and, A = Anterior. 1 = Temporal Cortex, 2 = Thalamus, 3 = Lateral Frontal Cortex, 4 = Temporal Cortex, 5 = Medial Frontal Cortex. The oblique lines in C and F represent data of each participant, while vertical lines denote the standard error (*** < 0.001, **<0.01).

To describe the brain correlates of human face perception in dogs, we contrasted the activity of human faces and everyday objects (human faces > objects), which revealed two clusters (Fig 3). The first cluster (37,420 mm^3^) was located in the left temporal cortex and extended to the frontal cortex, the caudate nucleus and the thalamus. The second cluster (30,404 mm^3^) was located in the right frontal cortex and extended to the right temporal cortex (see S1 Fig and S1 Table for information about the location and BOLD signal change within local maxima of these clusters). All dogs showed a clearly different BOLD response to each stimulus category, which is evident from the data extracted from a sphere centered at the peak *z* value in each resulting cluster (Fig 3C and 3F).

**Fig 4:**
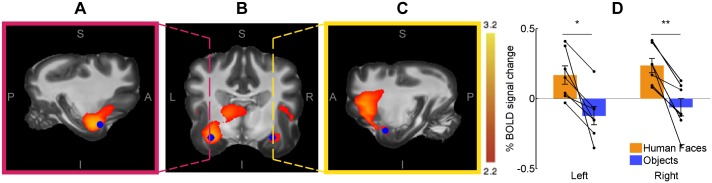
Bilateral temporal cortex in the contrast Human faces > Objects (n = 7). The cerebral activity focused in the temporal cortex is shown overlaid on the Datta atlas [24]. **A.** Left hemisphere in a sagittal slice. **B.** Bilateral temporal cortex in a coronal slice. **C.** Right hemisphere in a sagittal slice. Dashed lines in B show the location of the sagittal slices shown in A and C. **D.** BOLD signal change in a sphere of 5 mm radius sphere centered at the voxel with maximum *z* value for the both hemispheres (blue spheres show in A, B and C). Oblique lines represent the data of each participant and vertical lines represent standard error. * < 0.05; ** < 0.01.

The temporal cortex showed bilateral activation in the contrast human face > objects, as shown in Fig 4. The left temporal lobe showed a greater extent of activation than the right temporal lobe. However, an analysis of BOLD signal of a spherical region (5 mm radius) in two homologous regions inside the temporal activations showed no inter-hemispheric asymmetry of the differential responses to faces and objects (p = 0.95). The opposite contrast (objects > human faces) showed no significant differences, even when the *z* value threshold was decreased to 1.6.

The peri-stimulus time course of the BOLD signal was extracted from a sphere (5 mm radius) centered at the voxel with the maximum *z* value for the contrast face > objects within the temporal cluster in each participant (blue sphere in Fig 3A and 3B). The average of all blocks for all participants showed significant differences between human faces and objects, 3.5 seconds after the start of the block; this difference was maintained for 7 seconds (S2 Fig). Interestingly, the hemodynamic response was modulated by human faces and objects in opposite directions, with an increase in response to human faces as large as the decrease in signal in response to objects. The hemodynamic response to human faces is consistent with a previous study in dogs, with a peak in the response 3–5 seconds after the stimuli [27].

**Fig 5:**
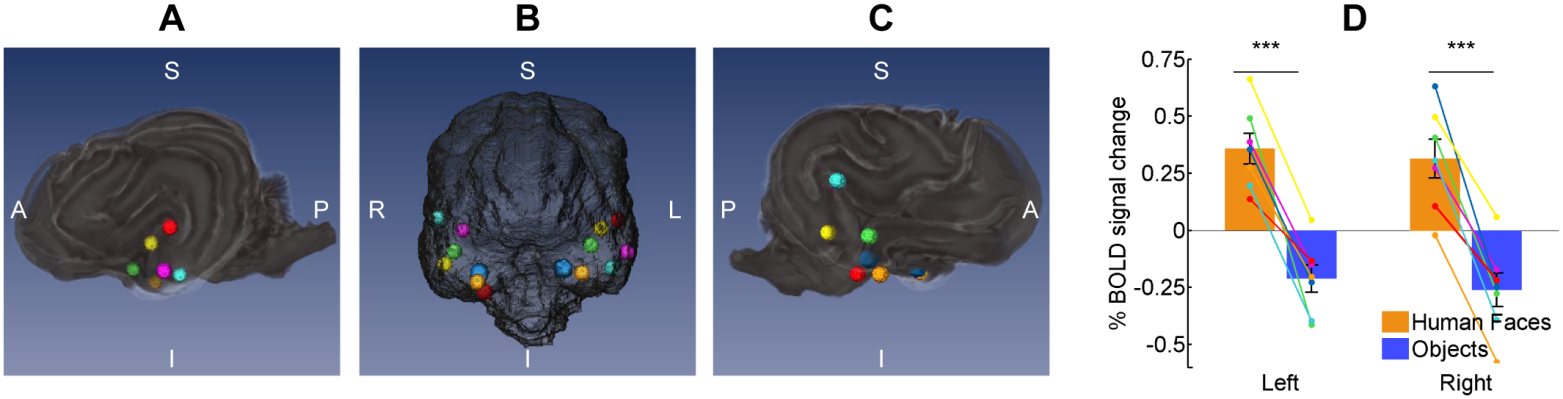
Location of the most sensitive region for faces in each participant in the temporal cortex. **A-C:** Left lateral, anterior and right lateral views showing spheres of 5 mm radius in each hemisphere, colored differently for each dog. **D:** BOLD signal change in both hemispheres in response to faces and objects within the sphere of each participant. Lines represent the data of each dog, colors according to spheres in A-C. Vertical lines represent the standard error (*** < 0.001).

At the subject level, there was variability in the location of regions most sensitive to faces (Fig 5). We specifically searched for these regions within the Sylvian gyrus bilaterally [24], and extracted BOLD signal change from spheres of 5 mm radius (see S2 Table for coordinates of each sphere). Despite slight anatomical variability, all dogs showed a very similar pattern of differential BOLD activity in response to each type of stimulus (Fig 5D).

In a separate analysis, we contrasted human faces > objects, but only within those voxels that had already shown a significant activity in response to any of the two types of visual stimuli, with respect to baseline. The cluster showing activity to any type of visual stimulus was large and, as expected, restricted to the occipital lobes (164,443 mm^3^). There were several regions within this large cluster that showed a differential activation favoring faces over objects (S3 Fig), but these were only seen on uncorrected statistical maps (thresholded at p_uncorr_ < 0.05), and these regions were not significant after correction for multiple comparisons.

## Discussion

The present study reveals fMRI evidence of cortical and subcortical activity related to visual processing of human faces in the dog brain, particularly within ventral-posterior regions of the temporal lobes (Figs 3 and 4). We suggest that this portion of the temporal cortex in dogs could be anatomically and functionally similar to regions found in other species, like humans [1], non-human primates [28] and sheep [29], which suggests a high degree of evolutionary conservation of the ventral visual pathway for face processing. The most consistent and robust face-selective activation in humans is located on the lateral side of the mid-fusiform gyrus [2] within the temporal lobe, which is in line with a recent report using similar methodology [19]. There are remarkable similarities in face processing in macaques and sheep, which suggest that they have a similar specialized mechanism for face perception localized in the temporal cortex [30]. Using fMRI, face-selective regions have been found in the inferior temporal lobe in macaques [28]. Similarly, electrophysiological studies have demonstrated that sheep have neuronal populations in the temporal cortex that respond preferentially to faces [30]. In the case of dogs, the Sylvian gyrus (ventral temporal cortex) displays robust activity to faces in comparison to objects. However, in comparison to studies performed on humans and non-human primates, the anatomical location of face sensitive regions we show in awake dogs is more variable amongst individuals (Fig 5); this variability could be due to anatomical or functional differences between subjects, but may also be due to the fact that primate studies have much higher signal-to-noise ratio than one achieved in this study. Humans, too, show some anatomic variability of FFA [31], albeit to a lesser degree than what we show in dogs. This variability was also noted in dogs in a recent study of face processing in dogs; in that study, similarly to our results, face processing regions were located within the temporal cortices in all participants [19]. It is interesting that in humans [1], non-human primates [28,32], sheep [29] and dogs, the processing of faces involves the temporal cortex, albeit not in the exact same location. Even across the primates, humans and macaques differ in terms of the location and number of regions sensitive to faces, suggesting a shift of the location of the face area throughout primate evolution [33]. While precise function of each patch sensitive to faces in the macaque brain is still unclear [33], the anterior medial patch is the only region that shows clear activity in response to faces shown in any angle [34] making it a likely homologue to FFA in humans. Future studies are needed to test whether an analogue of these regions exists in dogs.

As has been seen in humans [1], face processing in dogs revealed activity in several brain areas beyond the temporal lobe, including the medial and lateral frontal cortex. A previous fMRI study with dogs reported activity in the medial frontal cortex related to familiar scents, especially to a familiar dog scent [20]. It is possible that the activity in the medial frontal cortex reflects familiarity with, or salience of, the kind of stimulus presented. In our case, it may be that dogs were more familiar with a human face than with objects and thus, showed enhanced activity in the medial frontal cortex. Alternatively, activation of the medial frontal cortex may be due to a greater salience of faces as compared with objects. The activity in the lateral frontal region was found mainly, but not exclusively, in the right hemisphere. There are behavioral traits that are suggestive of right hemispheric preference related to novel [35] or emotionally charged stimuli, such as listening to a thunderstorm [36] or to human commands with intonational salience [37]. The faces we used in our paradigm were not familiar to the dogs and qualified as emotionally neutral, yet it is known that these faces can often be interpreted as negative by humans [38]; the right hemispheric preference seen in our results could be the due to the salience or valence assigned by dogs' perception of human faces. Nonetheless, we cannot rule out that, as with other species, the activity seen in the frontal cortex is related to other functional processes [39].

We also found activity in subcortical structures, namely in the caudate and the thalamus. The caudate activations are especially interesting, because this cerebral structure has been reported in several studies in dogs using fMRI. In the first study, the authors suggested that the caudate activation is associated with reward [27]. In a later study [18], the authors replicated the caudate activation and found that the activation was greater in service dogs when a human was showing them a sign related to a reward. In a different study, the caudate activation was related to a scent of familiar humans and dogs, but the maximum response was to the scent of a familiar human [20]. In this light, our data suggest that the caudate region is involved in reward processes, but not necessarily related to familiar stimuli. It is possible that, for a dog, observing a human face is intrinsically more rewarding than the sight of an object, in line with behavioral studies that have proven that dogs find human faces to be a salient stimulus [10]. The thalamus has been related in humans to an emotional response towards faces [40–42]. The face stimuli used in our study were neutral, although we don't have a way of knowing how dogs interpret faces and, given our current data, it is difficult to link the thalamic activity to a specific cognitive process.

There are other regions related to processing of faces in humans, such as the occipital face area and the posterior region of the superior temporal sulcus [1]. These regions are thought to be a set of stages in the hierarchical processing for face perception [43]. When we restricted our statistical analyses to regions active in response to visual stimuli (regardless their category) with respect to baseline, we found face-selective regions within the occipital lobes, (S3 Fig). However, these results were not corrected for multiple comparisons and we cannot conclude that this activity is similar to occipital face area seen in humans.

One limitation in our study is that the dogs passively viewed the stimuli; we minimized the potential of habituation by presenting each stimulus only twice throughout the experiment, but we cannot be certain if the dog has stopped paying attention. An open challenge is to receive an active behavioral response from the dogs without promoting movement. Also, we did not determine the spatial viewing pattern of images in our participants. There is evidence using eye tracking that dogs show significant differences in the number of fixations and total fixation duration between a blank screen and images, and dogs show a higher total duration time when observing a human in comparison to an object [44].

Given the importance of the faces for social behavior, we see this study as the beginning of new efforts to find the cerebral correlates of the perception and processing of faces in dogs. An important unclear issue is how dogs represent faces. In this study we focused only in human faces, mainly for the mutual attachment between dogs and humans [45,46] and because there is plenty of evidence that dogs can extract valuable information from human faces, like emotional state or identity [12,14]. The recognition of human faces in dogs may have been a crucial factor for their adaptation, given their natural anthropogenic niche, as some dogs can have more contact with human faces than with other dogs’ faces [12]. The recognition of human faces by dogs could be an essential factor for establishing attachment with humans. This is supported by the evidence found so far, that dogs, and no other canids, are able to recognize and attend to human faces without training [11]. Nonetheless, it is important to recognize that, given our data, we cannot determine if the temporal cortex is involved in the representations of faces in general, as we did not include faces of other species, including dogs. A recent study [19], however, suggests that the temporal cortex is involved in processing of faces of humans, as well as dogs; our results confirm and expand these findings showing that the temporal cortex, in conjunction with frontal cortex, thalamus and caudate, is involved in the perception of human faces. Moreover, our experimental design allowed for comparison of visual stimulation to baseline activity, allowing the identification of occipital regions suggestive of face processing.

Given that face recognition is present in all major vertebrate taxa, it is possible that visual species recognition emerged early in the evolution of mammals [47]. Besides, many species exhibit a conspecific advantage (the recognition and discrimination of members of their own species more readily than other species) [47], and it is therefore possible that face recognition of humans in dogs occurs in the same specialized neural and cognitive mechanisms used to process faces of its own species. We suggest that a general face processing system is localized in the temporal cortex, while categorization between species occurs in other cerebral regions, although (given the stimulation paradigm used) our present data cannot test this.

The comparison of the cognitive processes across different species will allow us to broaden our understanding of cognition and will help us construct a bottom-up perspective focused on the underlying cerebral mechanisms [48]. The use of dogs as a model allows us to explore fascinating topics of comparative social cognition, like the refinements produced by adaptations to an anthropogenic niche. Dogs have a predisposition to cooperate with humans [6] that, combined with non-invasive methodologies, make a perfect marriage for their use as a model. More than ten years ago Miklósi, Topál & Csányi wrote about "The beginning of an adventure" [7], and thanks to them and other groups, we know more today about our best friend. This adventure now includes information derived from functional imaging studies with awake and unrestrained dogs, as pioneered by Berns, Brooks & Spivak [27]. We hope that our study contributes to a better understanding of one of the foundations of social cognition in dogs: the perception of human faces.

## Supporting Information

S1 Fig BOLD signal change in the local maxima of each of the two clusters resulting from the contrast faces > objects. The BOLD signal change was extracted from a sphere of 5 mm of radius around each local maxima (see S1 Table for corresponding spatial information). **A.** Local maxima within the Temporal cluster. **B.** Local maxima within the Frontal cluster. The vertical lines represent the standard error (* < 0.05, ** < 0.01; *** < 0.001).
(TIF)

S2 Fig Peri-stimulus BOLD time course for faces and objects in temporal cortex (n = 7). The responses of all blocks for all participants were averaged. Dotted lines mark the start and end of the stimulation block. There are significant differences in the response between human faces and objects between 3.5 and 10.5 seconds after the presentation of the stimuli. * < 0.05, ** < 0.01; *** < 0.001.
(TIF)

S3 Fig Human faces > Objects in visual areas (n = 7). Volume renderings of the Datta atlas (grayscale) and statistical parametric maps. Hot colors indicate the regions showing increased BOLD activity in response to visual stimuli (regardless of category), with respect to baseline. Cool colors show regions within the already identified visual areas that showed a differential activation favoring faces over objects, thresholded at p_uncorr_ < 0.05. **A.** Posterior view. **B.** Superior view. **C.** Lateral view of the left hemisphere. **D.** Lateral view of the right hemisphere.
(TIF)

S1 Table Local Maxima of the temporal and frontal clusters resulting from the Contrast Faces > Objects. Coordinates are given according to the Datta atlas [24].
(PDF)

S2 Table Localization of the center of individual spheres in the Sylvian gyrus. Coordinates are given in mm, according to Datta atlas [24].
(PDF)
