# Supplementary figures and images for: Our Faces in the Dog's Brain: Functional Imaging Reveals Temporal Cortex Activation during Perception of Human Faces
Source: PLoS One. 2016 Mar 2;11(3):e0149431. doi: 10.1371/journal.pone.0149431 (PMC4774982; doi:10.1371/journal.pone.0149431)

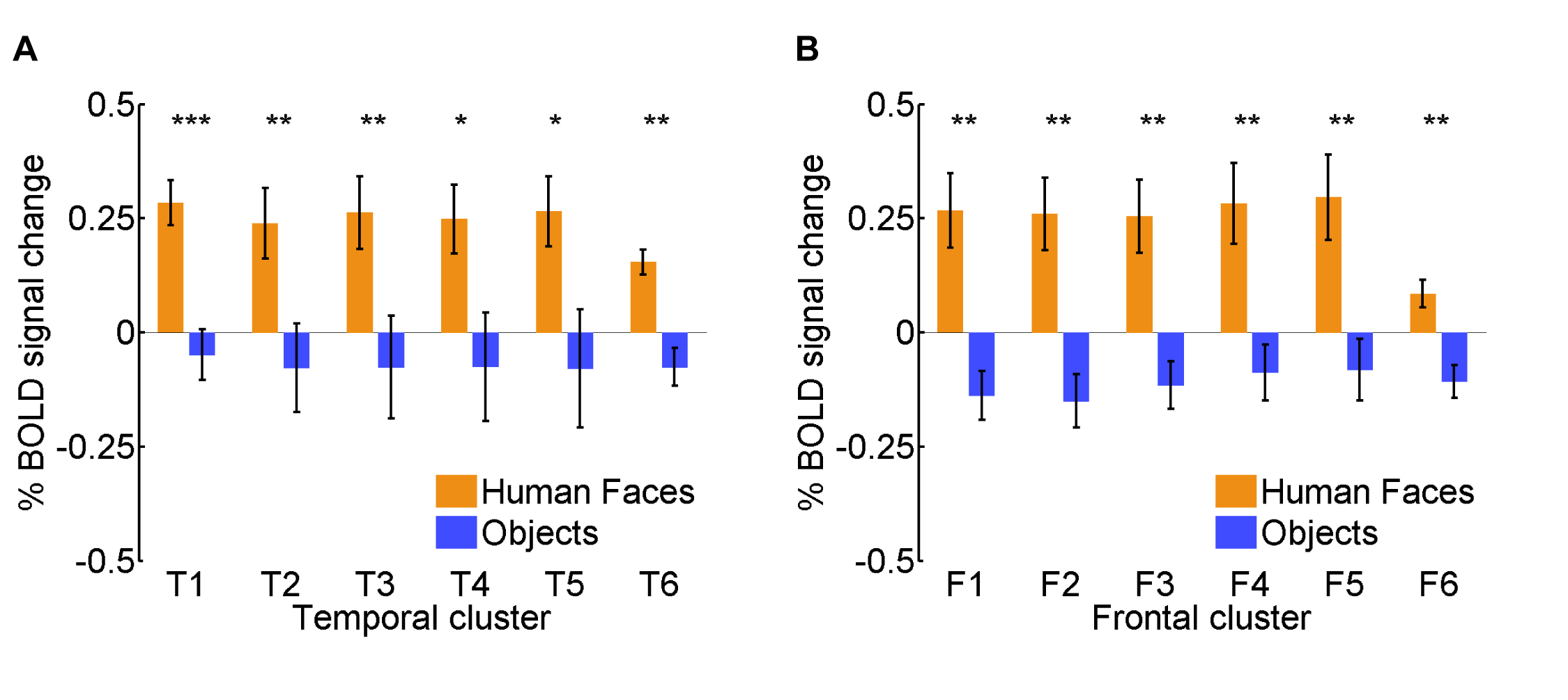

Supplement: S1 Fig — The BOLD signal change was extracted from a sphere of 5 mm of radius around each local maxima (see S1 Table for corresponding spatial information). A. Local maxima within the Temporal cluster. B. Local maxima within the Frontal cluster. The vertical lines represent the standard error (* < 0.05, ** < 0.01; *** < 0.001). (TIF) [file pone.0149431.s001.tif]

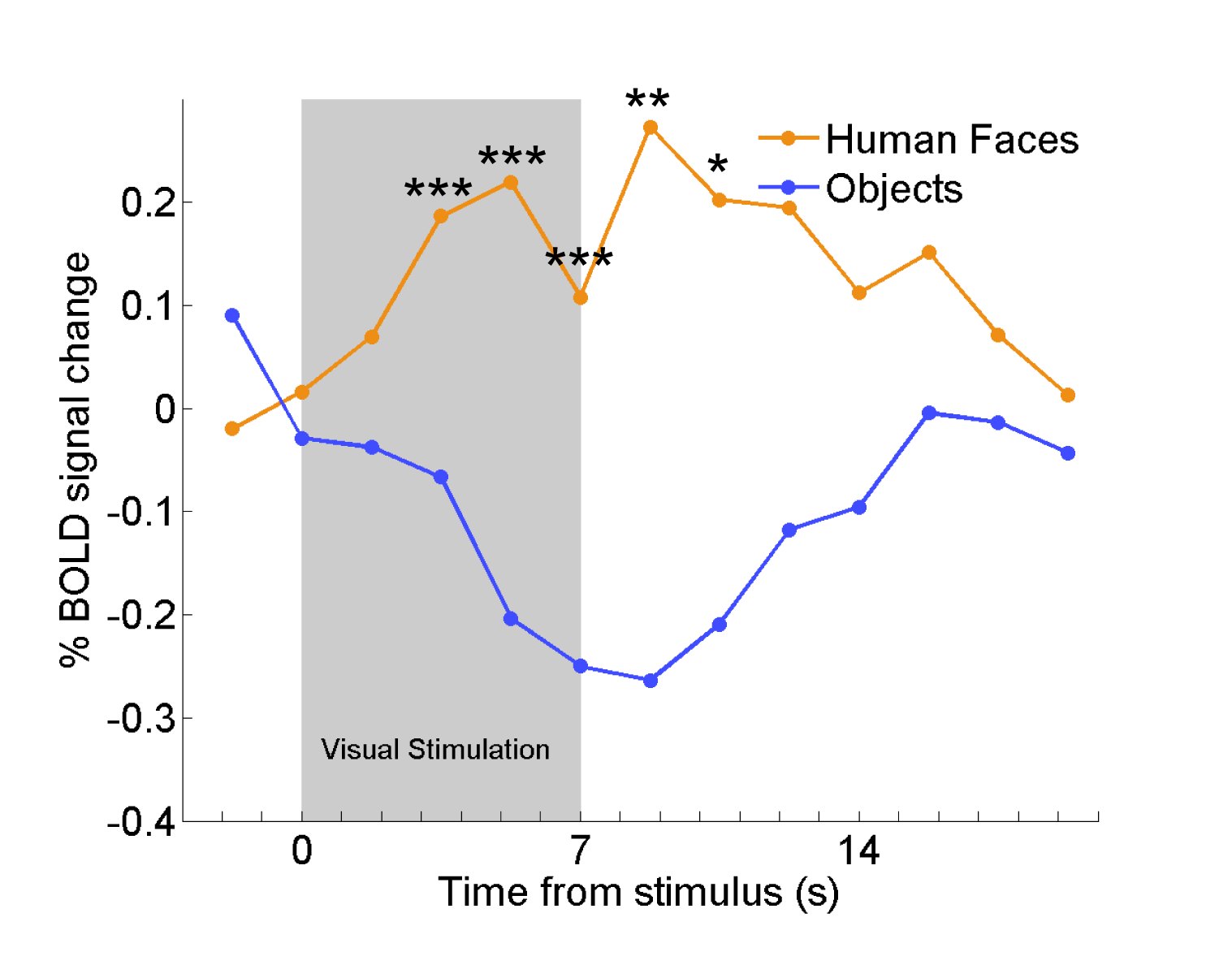

Supplement: S2 Fig — The responses of all blocks for all participants were averaged. Dotted lines mark the start and end of the stimulation block. There are significant differences in the response between human faces and objects between 3.5 and 10.5 seconds after the presentation of the stimuli. * < 0.05, ** < 0.01; *** < 0.001. (TIF) [file pone.0149431.s002.tif]

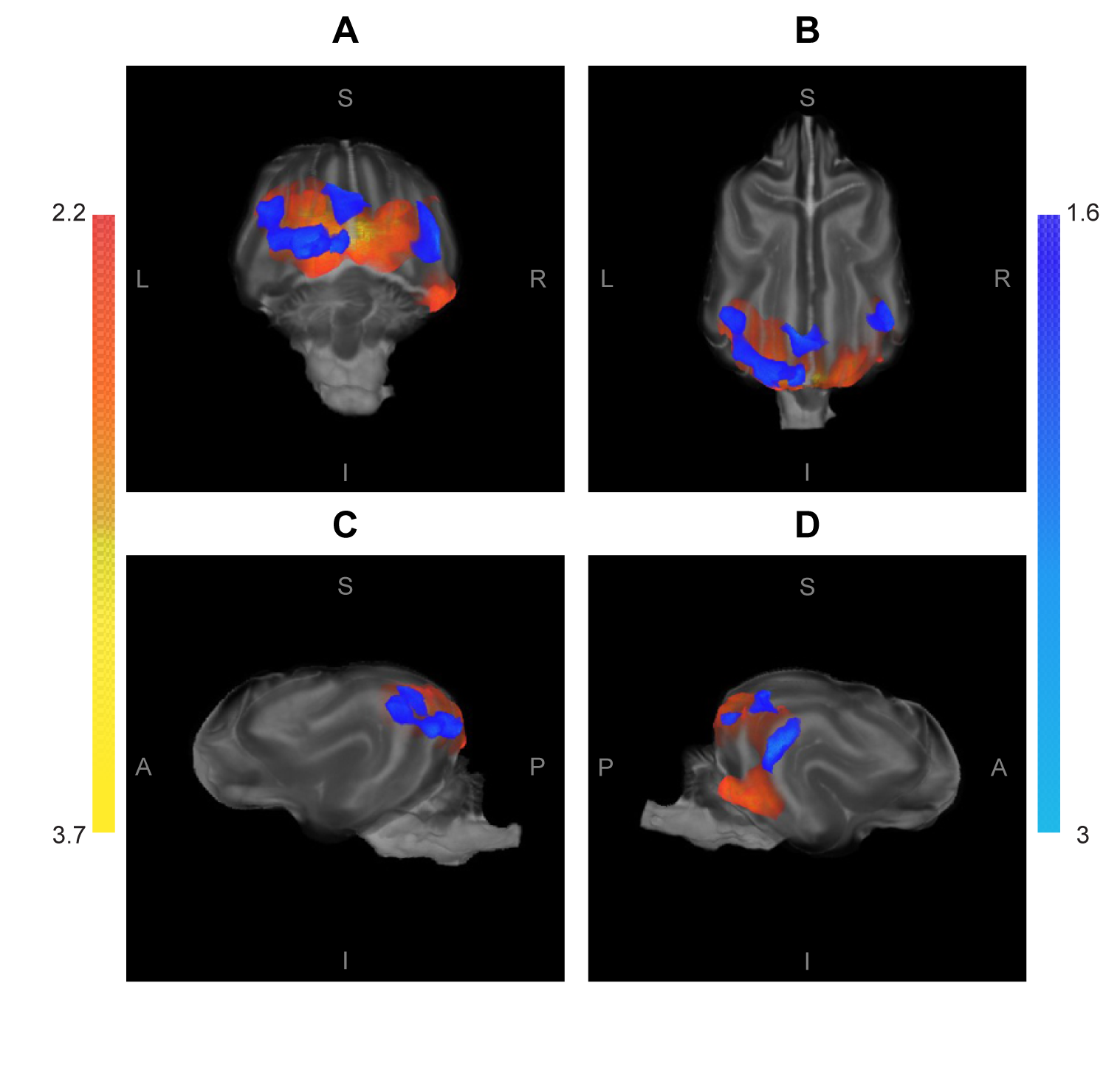

Supplement: S3 Fig — Volume renderings of the Datta atlas (grayscale) and statistical parametric maps. Hot colors indicate the regions showing increased BOLD activity in response to visual stimuli (regardless of category), with respect to baseline. Cool colors show regions within the already identified visual areas that showed a differential activation favoring faces over objects, thresholded at puncorr < 0.05. A. Posterior view. B. Superior view. C. Lateral view of the left hemisphere. D. Lateral view of the right hemisphere. (TIF) [file pone.0149431.s003.tif]
