# Supplementary material for: Our Faces in the Dog's Brain: Functional Imaging Reveals Temporal Cortex Activation during Perception of Human Faces
Source: PLoS One. 2016 Mar 2;11(3):e0149431. doi: 10.1371/journal.pone.0149431 (PMC4774982; doi:10.1371/journal.pone.0149431)
Supplement: S1 Table — Coordinates are given according to the Datta atlas [24]. (PDF) [file pone.0149431.s004.pdf]

Cluster	Local Maxima	Z-Max	X	Y	Z
Temporal	T1	2.96	93	139	102
	T2	2.8	127	186	112
	T3	2.76	132	184	112
	T4	2.75	135	182	111
	T5	2.74	132	181	111
	T6	2.73	118	133	135
Frontal	F1	2.87	170	179	139
	F2	2.87	170	182	140
	F3	2.83	172	173	139
	F4	2.81	169	172	134
	F5	2.81	168	172	131
	F6	2.75	158	162	156
