# Supplementary material for: Our Faces in the Dog's Brain: Functional Imaging Reveals Temporal Cortex Activation during Perception of Human Faces
Source: PLoS One. 2016 Mar 2;11(3):e0149431. doi: 10.1371/journal.pone.0149431 (PMC4774982; doi:10.1371/journal.pone.0149431)
Supplement: S2 Table — Coordinates are given in mm, according to Datta atlas [24]. (PDF) [file pone.0149431.s005.pdf]

	Left hemisphere					Right hemisphere			
Participant	Z-Max	X	Y	Z		Z-Max	X	Y	Z
Hera	3.3	89	154	111		2.6	182	152	108
Kora	3.8	63	130	113		3.5	182	112	136
Kun-kun	2.6	108	146	97		3.9	165	143	95
Morante	3.1	81	135	129		3.8	192	127	106
Morris	3.4	95	142	95		3.2	165	154	86
Odín	2.5	67	118	141		2.6	162	134	84
Zilla	3.3	73	121	104		3.4	198	131	139
